# Epidemiologic Features of Kawasaki Disease in Japan: Results of the 2009–2010 Nationwide Survey

**DOI:** 10.2188/jea.JE20110126

**Published:** 2012-05-05

**Authors:** Yosikazu Nakamura, Mayumi Yashiro, Ritei Uehara, Atsuko Sadakane, Satoshi Tsuboi, Yasuko Aoyama, Kazuhiko Kotani, Enkh-Oyun Tsogzolbaatar, Hiroshi Yanagawa

**Affiliations:** Department of Public Health, Jichi Medical University, Shimotsuke, Japan

**Keywords:** mucocutaneous lymph node syndrome, incidence, cardiovascular diseases, immunoglobulin, intravenous, epidemiology

## Abstract

**Background:**

Although the number of patients and incidence rate of Kawasaki disease (KD) are increasing in Japan, the most recent epidemiologic features of KD are not known.

**Methods:**

The 21st nationwide survey of KD was conducted in 2011 and included patients treated for the disease in 2009 and 2010. Hospitals specializing in pediatrics, and hospitals with a total of 100 or more beds and a pediatric department, were asked to report all patients with KD during the 2 survey years.

**Results:**

A total of 1445 departments and hospitals reported 23 730 KD patients (10 975 in 2009 and 12 755 in 2010): 13 515 boys and 10 215 girls. The annual incidence rates were 206.2 and 239.6 per 100 000 children aged 0 to 4 years in 2009 and 2010, respectively; the 2010 rate was the highest ever reported in Japan. Monthly number of patients peaked during winter to spring months; lower peaks were noted during summer months. However, the seasonal patterns in 2009 and 2010 differed from those of previous years. The age-specific incidence rate had a monomodal distribution, with a peak during the latter half of the year of birth. The prevalences of cardiac lesions during acute KD and cardiac sequelae were higher among infants and older age groups. Despite a decrease in prevalence, the proportion of patients with giant coronary aneurysms—the most severe sequela of KD—did not substantially decrease.

**Conclusions:**

The incidence rate and number of patients with KD continue to increase in Japan.

## INTRODUCTION

Kawasaki disease (KD) is a syndrome of unknown etiology. It typically affects infants and toddlers and causes systemic vasculitis.^1^^,^^2^ Cardiac lesions, eg, coronary artery aneurysms, are a hallmark of the disease,^3^^–^^7^ although the proportion of patients with such lesions at 1 month after onset is now less than 5%. The most serious cardiac lesions are giant coronary aneurysms (diameter ≥8 mm on 2-dimensional echocardiography), which are associated with an unfavorable prognosis. Prevention of these aneurysms is the primary target for pediatricians treating patients with KD.

Since 1970, nationwide epidemiologic surveys of KD have been conducted in Japan nearly every 2 years, and several features of the disease have been revealed.^8^^–^^13^ The most recent previous survey, the 20th, included patients treated in 2007 and 2008 and showed that the annual number of patients and the incidence rate had increased linearly. In 2011 we conducted this 21st survey, which targets patients treated in 2009 and 2010.

Herein, we report the results of the latest nationwide survey of KD patients treated in 2009 and 2010 in Japan.

## METHODS

We conducted a retrospective survey of patients with KD who visited target hospitals for treatment of acute KD during the 2-year period from January 2009 through December 2010. The medical facilities that were requested to participate in the survey were hospitals specializing in pediatrics and hospitals with a total of 100 or more beds and a pediatric department. These criteria have been used since the first nationwide survey, in 1970.^14^ Questionnaires (http://www.jichi.ac.jp/dph/kawasakibyou/20100715/kawasaki21final20100715.pdf, in Japanese) and diagnostic guidelines prepared by the Japan Kawasaki Disease Research Committee^15^ were sent by mail to administrators in charge of the pediatric department of their respective hospitals in January 2011. The prepared list of hospitals for the survey was based on the *Listing of Hospitals 2003–2004* compiled by the Committee on Studies of Health Policies, Ministry of Health, Labour and Welfare, Japan and was revised using newly received information. A total of 2072 facilities met the conditions stated above.

Patient information requested on the questionnaire was: name (initials only), address (municipality), sex, date of birth, birth conditions (weight at birth and gestational age), date and day of illness at first hospital visit, diagnosis (typical definite, atypical definite, or incomplete), intravenous immunoglobulin (IVIG) therapy, IVIG resistance status, additional therapy if conducted (additional IVIG therapy, steroids, infliximab, immunosuppressive agents, and plasmapheresis), recurrences, history of KD among patient’s siblings and parents, cardiac lesions, and complications other than cardiac lesions (ie, encephalitis/encephalopathy, severe cardiomyositis requiring treatment, arrhythmia with tachycardia, vomiting/diarrhea, bronchitis/pneumonia, and macroscopic hematuria). Acute cardiac lesions were defined as those that developed within 1 month of onset (acute lesions); cardiac sequelae were defined as those that persisted beyond 1 month after onset. Almost all patients were diagnosed on the basis of 2-dimensional echocardiography.

After checking for possible inconsistencies on the questionnaires, the forms were sent back to the respondents to correct any errors. The incidence rates were based on the population data used in the vital statistics of Japan.^16^ The Ethical Board of Jichi Medical University approved this survey in advance (August 31, 2010, No. 10-23).

## RESULTS

Of the 2072 invitations sent requesting participation in the survey, 39 were returned because the pediatric department, or the institution itself, had closed. Of the remaining 2033 departments, 1445 (71.1%) responded to the survey and reported a total of 23 730 patients (10 975 in 2009 and 12 755 in 2010). There were 13 515 male patients and 10 215 female patients. The average annual incidence rate for the observed 2-year period was 222.9 per 100 000 children aged 0 to 4 years (247.6 for boys and 196.9 for girls).

**Figure 1.**
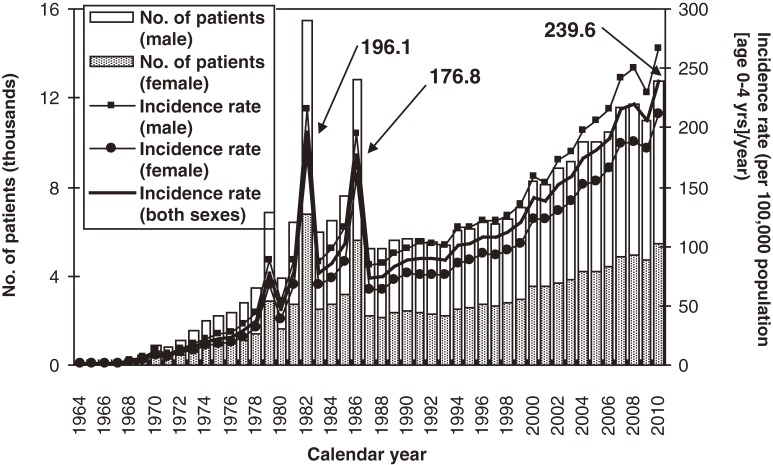
Number of patients with Kawasaki disease and incidence rate in Japan, by calendar year

The annual numbers of patients with KD and the incidence rates in the 21 nationwide surveys, including this one, are shown in Figure 1. As previously reported, there were 3 large nationwide epidemics of the disease in Japan, in 1979, 1982, and 1986. Since then, there has been no nationwide epidemic, but the number of patients started to increase in the mid-1990s. Because of the decrease in the birth rate in Japan, the incidence rate increased more rapidly than the number of patients, reaching 239.6 per 100 000 children aged 0 to 4 years in 2010. This was the first time the incidence rate exceeded 230, and it surpassed the rates observed in 1979, 1982, and 1986, when nationwide epidemics occurred.

**Figure 2.**
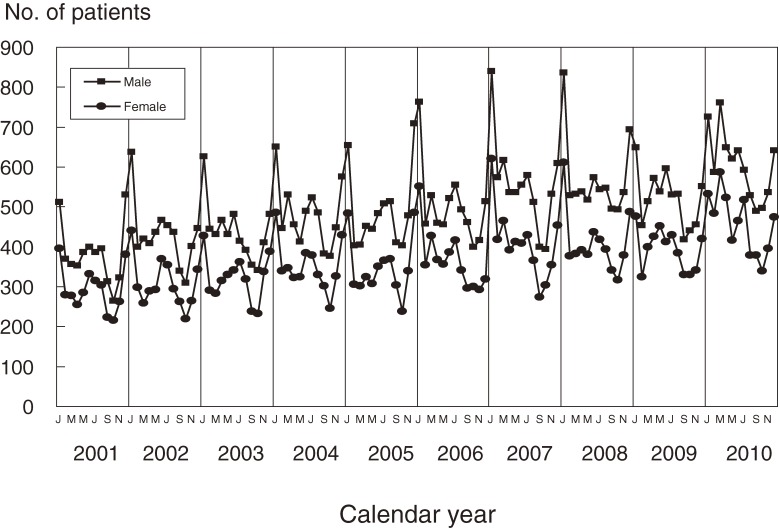
Number of patients with Kawasaki disease in Japan by month, 2001–2010

Figure 2 shows trends in the monthly number of patients observed in the 5 most recent nationwide surveys (17th to 21st). Seasonal patterns in the latest survey slightly differed from those in previous surveys, although the basic pattern—a maximum during the winter and a lower peak in the summer—remained consistent. The 2009 and 2010 winter peaks were lower than in previous years. The peak in March 2010, which was new for the most recent 10-year period, was higher than that in January.

**Figure 3.**
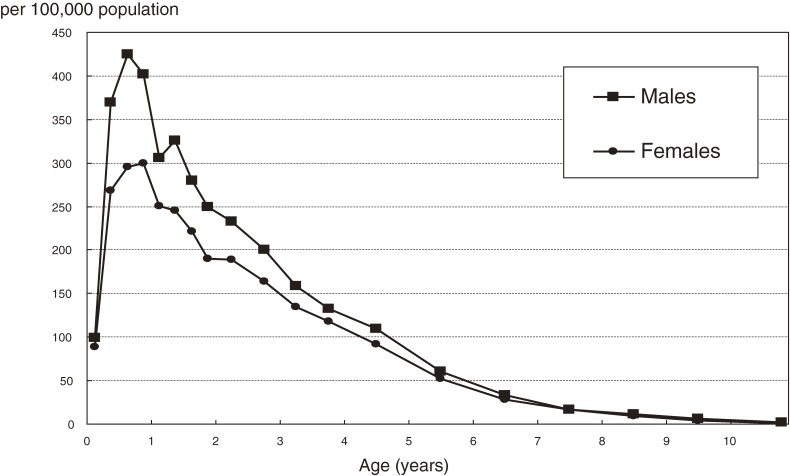
Age-specific annual incidence rate of Kawasaki disease in Japan, 2009–2010

Age-specific incidence rates by sex are shown in Figure 3. As in previous surveys, the incidence rate was highest among children aged 6 to 11 months, after which it gradually decreased with advancing age, with the exception of an increase at age 1 year among males.

Of the 23 730 patients reported, 18 680 (78.7%) were typical definite cases (patients with 5 or 6 of the symptoms specified in the diagnostic guidelines for KD), 618 (2.6%) were atypical definite cases (4 of the 6 symptoms plus coronary aneurysms including dilatation), and 4410 (18.6%) were incomplete cases (patients who did not satisfy the diagnostic criteria but were suspected as having KD by the pediatricians reporting the cases). Of the 4410 incomplete cases, 2894 (65.6%) had 4 of the 6 principal symptoms, 1175 (26.6%) had 3 symptoms, 271 (6.1%) had 2, and 30 (0.7%) had 1.

The number of patients with 1 or more siblings affected by KD was 376 (1.6%); 163 (0.7%) patients had at least 1 parent with a history of KD. There were 848 (3.6%) recurrent cases. Of the 23 730 patients reported, 1 died of cerebral infarction at age 3 months.

Gestational age and body weight at birth were recorded for 9346 patients who were born between 22 and 43 weeks of gestational age: 8507 (91.0%) were born at term, and 769 (8.2%) were born preterm, including 36 who were born before 28 weeks’ gestation. Low birth weight (<2500 g) was reported for 578 (10.7%) males and 518 (13.2%) females, including 55 boys and 56 girls with a birth weight less than 1500 g.

**Figure 4.**
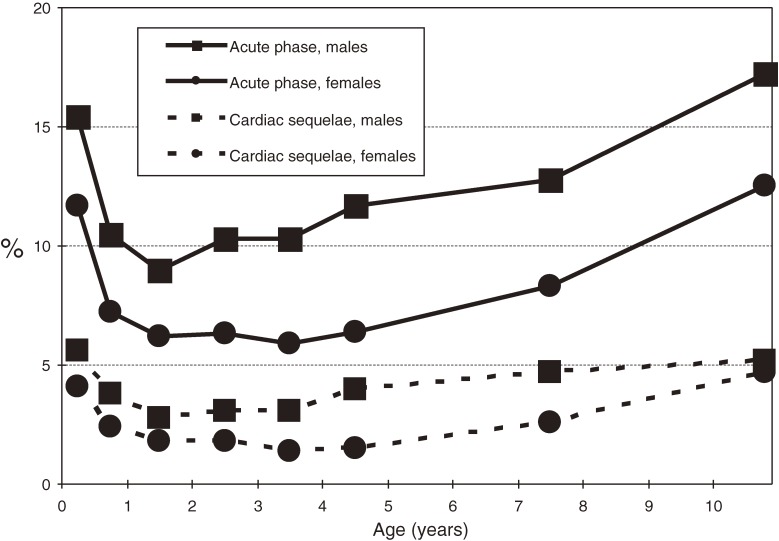
Age-specific prevalence of cardiac lesions and sequelae of Kawasaki disease in Japan, 2009–2010

During the acute phase, 2212 (9.3%) patients had 1 or more cardiac lesions: 58 (0.24%) had giant coronary aneurysms, 247 (1.04%) had coronary aneurysms less than 8 mm in diameter, 1722 (7.26%) had coronary dilatations, 7 (0.03%) had coronary stenoses, 2 (0.01%) had myocardial infarctions, and 283 (1.19%) had valvular lesions. A total of 711 patients (3.0%) had cardiac sequelae at 1 month after onset of KD: 53 (0.22%) had giant coronary aneurysms, 186 (0.78%) had coronary aneurysms less than 8 mm in diameter, 450 (1.90%) had coronary dilatations, 7 (0.03%) had coronary stenoses, 4 (0.02%) had myocardial infarctions, and 68 (0.29%) had valvular lesions. As shown in Figure 4, cardiac abnormalities were more prevalent in boys than in girls. They were also more prevalent in infants and older children than in children aged 1 to 4 years.

Of the 23 730 patients, 22 (0.09%) had encephalitis/encephalopathy, 38 (0.16%) had severe cardiomyositis requiring treatment, 17 (0.07%) had arrhythmia with tachycardia, 1045 (4.4%) had vomiting/diarrhea, 613 (2.6%) had bronchitis/pneumonia, and 10 (0.04%) had macroscopic hematuria.

Of the patients reported, 21 247 (89.5%) received IVIG therapy. Of these, 18 303 (86.2%) received IVIG therapy on or before the sixth day of illness. However, 3532 (16.6%) of the 21 247 patients treated with IVIG did not respond to the therapy (ie, fever continued despite therapy), and 3231 (91.5% of nonresponders) received additional IVIG therapy—1025 (29.0%) with steroids, 151 (4.3%) with infliximab, 129 (3.7%) with immunosuppressants, and 79 (2.2%) with plasmapheresis (the percentages sum to greater than 100% because some patients received more than 1 therapy).

## DISCUSSION

We presented the results of the 21st Nationwide Survey of Kawasaki Disease in Japan, which highlighted the most recent epidemiologic features of the disease. Since 1970, nationwide surveys have been conducted almost every 2 years.^8^^–^^13^ As shown in Figure 1, the number of patients and the incidence rate have rapidly increased since the mid-1990s. Even though there has been no nationwide epidemic of KD since 1986, the annual incidence rates in 2009 and 2010 were higher than those in the years of nationwide epidemics, with a small decrease in 2009. Because the etiology of KD remains unknown, the reasons for these increases are also unclear. This increase is of concern and highlights the need for continued observation of the epidemiologic features of KD in Japan. Moreover, the results should motivate researchers to hasten their efforts to identify the cause of this disease.

The response rate of the survey was 71.1%, after 2 reminders were sent. Therefore, the actual number of patients was higher than the reported figures. However, as discussed in a previous report, data suggest that the real figures are no more than 10% higher than the values reported herein.^17^ In addition, due to the catastrophic earthquake and tsunami of March 11, 2011,^18^ we delayed sending a reminder to hospitals in the stricken area; thus, the response rate was a little lower than that of the previous survey. Nevertheless, the survey response rate has remained approximately 70% during the most recent 10 years.

Although the epidemiologic features of KD in Japan, as revealed by this survey, were very similar to those shown in previous surveys, the seasonal patterns in 2009 and 2010 were slightly different, as shown in Figure 2. The peaks in January were not as high as in previous years, and in 2010 the monthly number of patients was highest in March. In 2009, a new influenza strain (H1N1) was prevalent worldwide, including Japan.^19^ The influenza epidemic might have changed the epidemic pattern of KD. However, the KD pattern in 2010, during which there was no epidemic of new influenza, also differed from that during and before 2008. The peak in January 2009, when H1N1 was not present, was not very high. The change in seasonal pattern thus cannot be completely explained by the influenza epidemic, although the influenza epidemic might have had an effect. Continued observation is necessary to determine whether the seasonal pattern has indeed changed or whether the patterns observed during 2009 and 2010 were specific to those years.

The proportion of typical definite cases was 78.7%, a decrease from 82.1% in the 16th survey (1999–2000),^20^ and the percentage of incomplete cases increased from 13.8% to 18.6%. These findings might reflect greater awareness of incomplete KD, and thus improved diagnosis, due to an increase in the number of pediatricians who are familiar with KD.

This was the first epidemiologic survey of KD in Japan to collect information on birth conditions (ie, gestational age and body weight at birth). One reason why data from only 39.4% (9346) of patients were collected is the prevalence of electronic medical record systems. When paper records were used in hospitals, pediatricians customarily recorded patient birth conditions, including gestational age and birth weight. However, many hospitals now use electronic records that lack space for such data. Vital statistics in Japan in 2009^21^ show that 5.7% of all births were preterm, 93.9% were term, and 0.4% were postterm. In addition, 8.5% of boys and 10.8% of girls were born with a body weight less than 2500 g. In comparison with the general population, patients with KD tend to be born earlier and with a lower birth weight.

One of the most serious problems in KD is cardiac sequelae, eg, coronary aneurysms and dilatation, although the proportion of such sequelae has decreased, from 6% in the 16th nationwide survey (1999 and 2000)^14^ to 3.0% in the current survey. Advances in managing patients during the acute phase, such as 2-dimensional echocardiography and IVIG treatment, likely contributed to this improvement. However, the proportion of patients with giant coronary aneurysms, the most serious sequela of KD, did not substantially decrease (0.40% in the 16th survey vs 0.22% in the current survey). This is the most immediate concern in KD management.

This is the first nationwide KD survey to report the proportions of complications other than cardiac lesions, such as encephalitis/encephalopathy and cardiomyositis. Although some of these complications are listed in diagnostic guidelines,^15^ their frequency has not been clear. The proportion of patients developing encephalitis/encephalopathy was 0.09% (average number of patients per year: 11), which might be higher than previously thought among Japanese pediatricians.

With regard to the 3 perspectives of descriptive epidemiology—person, place, and time—the epidemiologic features of KD revealed by Japanese nationwide surveys suggest the involvement of 1 or more infectious agents in KD occurrence. Regarding the personal factor, the existence of sibling cases of KD^22^^,^^23^ supports the infection hypothesis. In addition to the fact that 1.6% of reported patients in the current survey were sibling cases, previous studies showed that the incidence rate of KD among those having at least 1 sibling with the disease was approximately 10 times that of the general population and that the distribution of the period of time between KD onset in sibling pairs was concentrated on the same day and at 7 days.^22^ With regard to this latter finding, it is reasonable to assume that same-day onset reflects simultaneous infection and that the 7-day period is the incubation period for KD.

Although geographic movement of KD was not clear in recent nationwide surveys, epidemiologic data from the epidemic years of 1979, 1982, and 1986 do show such movement,^14^ which also supports the infectious agent hypothesis. The presence of seasonal variation, which differs between this and previous surveys, also supports the hypothesis. If KD is stimulated by several infectious agents that have different seasons of peak prevalence, numerous different peaks of KD could occur in a year. In addition, the seasonality of KD was not identical among the several countries and areas that have been investigated,^24^^,^^25^ which might be due to the presence of different causative agents among these countries/areas.

In conclusion, the 21st nationwide survey of KD in Japan showed that the number of patients and the incidence rate of KD continued to increase in 2009–2010. In addition, the accumulated epidemiologic data suggest an infectious agent(s) might trigger the onset of KD.
